# Measuring Coverage in MNCH: Tracking Progress in Health for Women and Children Using DHS and MICS Household Surveys

**DOI:** 10.1371/journal.pmed.1001391

**Published:** 2013-05-07

**Authors:** Attila Hancioglu, Fred Arnold

**Affiliations:** 1 Statistics and Monitoring Section, Division of Planning and Strategy, UNICEF, New York, New York, United States of America; 2 MEASURE DHS, ICF International, Calverton, Maryland, United States of America; Professor of Demography and Social Statistics, University of Southampton, United Kingdom

## Abstract

Household surveys are the primary data source of coverage indicators for children and women for most developing countries. Most of this information is generated by two global household survey programmes—the USAID-supported Demographic and Health Surveys (DHS) and the UNICEF-supported Multiple Indicator Cluster Surveys (MICS). In this review, we provide an overview of these two programmes, which cover a wide range of child and maternal health topics and provide estimates of many Millennium Development Goal indicators, as well as estimates of the indicators for the Countdown to 2015 initiative and the Commission on Information and Accountability for Women's and Children's Health. MICS and DHS collaborate closely and work through interagency processes to ensure that survey tools are harmonized and comparable as far as possible, but we highlight differences between DHS and MICS in the population covered and the reference periods used to measure coverage. These differences need to be considered when comparing estimates of reproductive, maternal, newborn, and child health indicators across countries and over time and we discuss the implications of these differences for coverage measurement. Finally, we discuss the need for survey planners and consumers of survey results to understand the strengths, limitations, and constraints of coverage measurements generated through household surveys, and address some technical issues surrounding sampling and quality control. We conclude that, although much effort has been made to improve coverage measurement in household surveys, continuing efforts are needed, including further research to improve and refine survey methods and analytical techniques.

## Abstract 2

In a *PLOS Medicine* Review, Attila Hancioglu and Fred Arnold describe the Demographic and Health Surveys (DHS) and Multiple Indicator Cluster Surveys (MICS) and highlight the methodological principles and challenges involved in using household survey data to measure reproductive, maternal, newborn, and child health intervention coverage.

*This paper is part of the* PLOS Medicine “*Measuring Coverage in MNCH" Collection*.

## Introduction

Considerable progress has been made since the mid-1990s in reducing maternal and child mortality [1]. However, there are still many unnecessary deaths among women and children (about 287,000 maternal deaths and 7.6 million child deaths in 2010 [2]), even though effective and affordable interventions are available. Tracking coverage indicators for women and children is necessary to guide global, regional, and country efforts to improve health so that scarce resources are directed to where they are most needed and will be most effective in saving lives.

Coverage measurements reflect the proportion of individuals needing an intervention, and must therefore be representative of the reference population. Two recent reviews assessed potential sources of coverage data for reproductive, maternal, newborn, and child health (RMNCH) interventions in the 75 countries that account for over 95% of maternal and child deaths [2],[3]. These reviews concluded that although routine data from health management information systems are the preferred source of coverage data because they provide information on a continuous basis at lower administrative levels such as districts, these systems are currently too weak in these countries to provide data of adequate quality for assessing and guiding health programmes. Demographic surveillance systems often produce higher quality data but for limited geographic areas that become progressively less representative of national populations over time if health intervention trials are conducted in these areas. Both reviews identified high-quality, nationally representative household surveys as the method of choice for measuring RMNCH coverage for the foreseeable future in most low- and middle-income countries. Importantly, even after health management information systems become reasonably complete and accurate, national household surveys will need to continue as a complementary data source since these surveys are representative of the general population and provide vital information on background characteristics and the determinants of population and health conditions. Household surveys are also needed for measuring inequalities in coverage [4].

A large majority of household surveys that have produced coverage estimates in low-income countries have been conducted under the USAID-supported Demographic and Health Surveys (DHS) [5] and the UNICEF-supported Multiple Indicator Cluster Surveys (MICS) programmes [6]. For example, in the global databases compiled by UNICEF, information on the use of oral rehydration therapy for children with diarrhoea comes from DHS and MICS surveys for 98% of the countries with available data. For care seeking for pneumonia, the comparable figure is 93%. DHS and MICS data have also made a major contribution to the scientific literature. According to a recent study and data from the DHS website, more than 1,100 articles based entirely or primarily on DHS data have been published in 346 peer-reviewed journals [7]. Both programmes provide free public access to survey reports and datasets.

Other standardized household survey programmes that have generated coverage data on selected indicators include the CDC-supported Reproductive Health Surveys conducted mostly in Latin America, Eastern Europe, and Central Asia between 1975 and 2009 [8], the Pan-Arab Population and Family Health Surveys (PAPFAM), supported by the Arab League and conducted in the Arab region [9], and the WHO-supported World Health Survey conducted from 2002–2004 [10]. There are also standard household surveys focused on individual diseases or intervention programmes such as the Malaria Indicator Surveys [11], the AIDS Indicator Surveys [12], and the Standardized Monitoring and Assessment of Relief and Transitions (SMART) surveys conducted in many sub-Saharan African countries in the early 2000s to provide information related to child nutrition, some of which are still being conducted on an annual or semi-annual basis [13]. Also, a few countries have mounted their own nationally representative surveys, usually based on adapted versions of the DHS and MICS protocols. Over time and as a result of arduous consultations at the global level, a set of standard indicators and “gold-standard" methodologies have emerged that are incorporated in DHS and MICS surveys, as well as in some more specialized surveys.

The methodological challenges of measuring RMNCH coverage received relatively little attention in the literature until recently, in contrast to the measurement of mortality [14]. Much of the testing of alternative coverage indicators, questions, and analytical techniques is available only in internal reports of work conducted by DHS or in the heads of the technical experts who have conducted the surveys. In this review, which is part of the *PLOS Medicine* “Measuring Coverage in MNCH" Collection, we draw on the DHS and MICS experience to highlight key methodological principles and challenges in using household survey data to measure RMNCH coverage. For more details, we direct readers to resource documents on survey design including sampling, questionnaires, data cleaning, and analysis as well as reports [5],[6],[15]. Other reviews and research articles in this collection will focus on measurement challenges in tracking trends in coverage for interventions targeting specific health conditions [16]–[21] and cross-cutting methodological issues [22]. Here, we complement this content by providing insights to help improve survey measurements of RMNCH coverage and to promote the informed use of coverage results to improve programmes.

## Overview of the DHS and MICS Survey Programmes

The DHS programme has been operating since 1984 with core funding from USAID and substantial contributions from other donors and participating countries. The programme is coordinated by ICF International. Its aim is to provide high-quality nationally representative data on health and population trends, with emphasis on fertility, family planning, mortality, reproductive health, child health, gender-related issues such as domestic violence, HIV/AIDS, malaria, and nutrition.

The MICS programme was developed by UNICEF in 1995 in response to the World Summit for Children and has expanded over time to measure progress towards the Millennium Development Goals and other international targets for women and children. MICS surveys provide key information on mortality, health, nutrition, education, HIV/AIDS, and child protection for use in programme decision making, advocacy, and national and global reporting.

**Figure 1:**
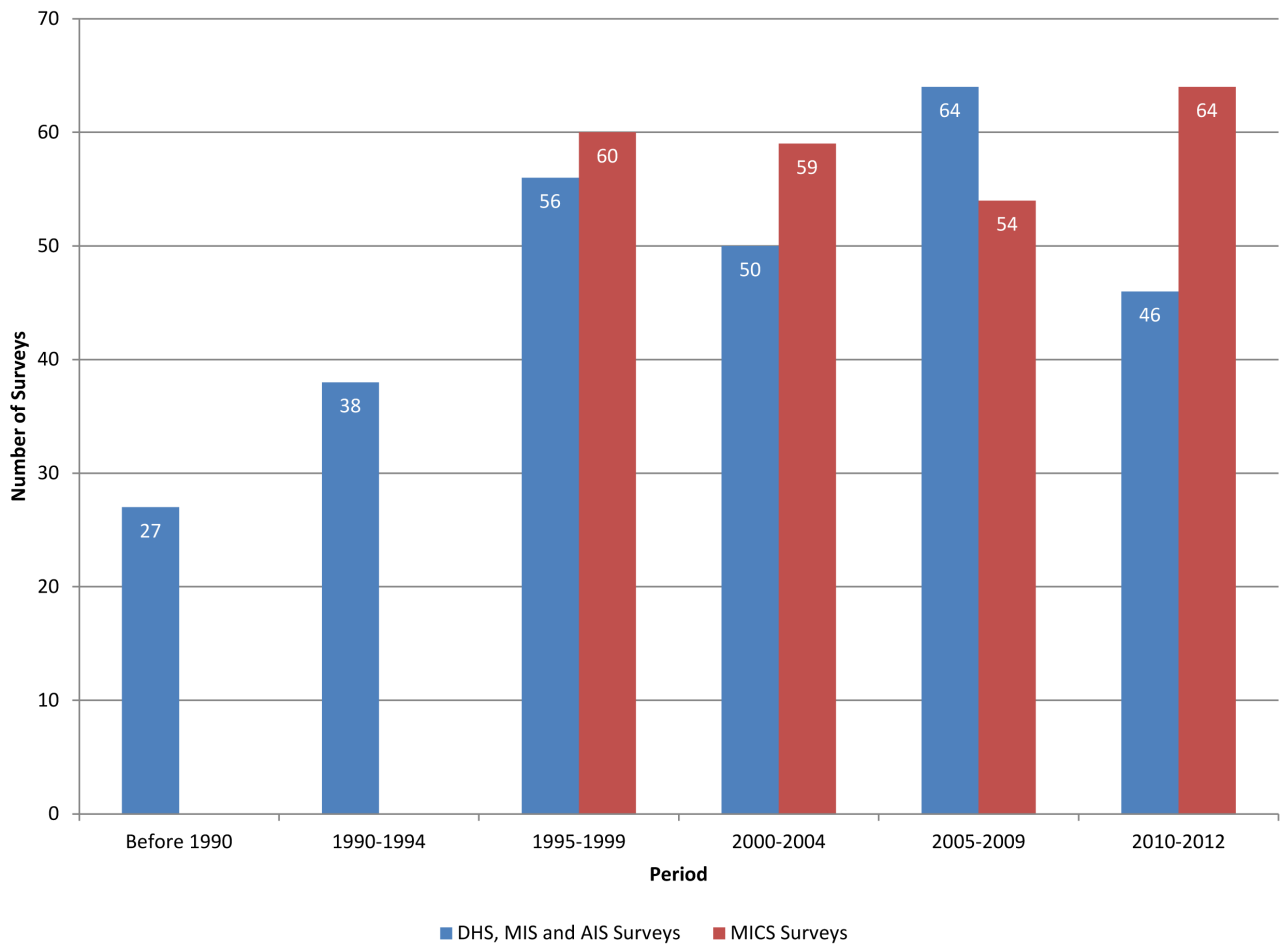
Number of DHS and MICS surveys by year.

Figure 1 shows the number of DHS and MICS surveys conducted annually since 1984. About ten to 15 DHS surveys have been conducted annually since 1995; consultative processes have led to major revisions in the core questionnaires every 5 years. MICS surveys were conducted in “rounds," every 5 years until 2007 and every 3 years thereafter, with about 60 surveys in each round.

The core DHS [23] and MICS [24] questionnaires have expanded over time, in content and complexity, to respond to country and global needs and the growing number of effective RMNCH interventions. Both surveys now include an increasing number of optional modules and complementary data collection tools for use in individual countries. Table 1 summarizes the characteristics of both survey programmes, including questionnaire content, which is typically decided through collaboration among government agencies, donors, key stakeholders, and DHS/MICS. Table 2 lists the major differences between DHS and MICS surveys and Box 1 highlights a particularly important difference between DHS and MICS surveys—the way in which they handle information on orphans and foster children.

**Table 1:** Characteristics of the DHS and MICS survey programmes.

Characteristics	DHS	MICS
**Content of “core" questionnaires and modules (2012)**		
***Both surveys***	Fertility and family planning; infant and child mortality; maternal mortality; antenatal care (number of visits, provider, components of antenatal care, intermittent preventive treatment for malaria during pregnancy); delivery care (place of birth, delivery assistance, cesarean section, birth weight, birth size); postnatal care (postnatal care visits, timing of visits, type of provider); child protection (birth registration, child marriage); child feeding practices (prelacteal feed, breastfeeding, diet); child immunisation coverage; childhood fever, acute respiratory infections, diarrhoea (prevalence, care-seeking behaviour, place and type of treatment); children's living arrangements; malaria (ownership and use of mosquito nets, treatment of fever, indoor residual spraying against mosquitoes, malaria diagnosis); HIV (knowledge of transmission and prevention, prior testing, stigma, and discrimination); sexual behaviour; female genital cutting; environmental health (water, sanitation, handwashing, disposal of children's stools, cooking fuel); biomarkers (height, weight)	See DHS
***Primarily one survey***	Vitamin A supplementation, iron supplementation, sexually transmitted infections other than HIV (self reports, symptoms), exposure to second-hand smoke, biomarkers including tests for anaemia, HIV, and malaria, timing of antenatal care visits, domestic violence, fistula, women's empowerment	Child labour, child discipline, early child development, knowledge of danger signs for child illness
***Complementary protocols (2012)***	MIS, AIS, SPA surveys, KIS	
**Guidelines for survey implementation**		
Length of interviewer training, including field practice	4 weeks	3 weeks
Composition of field teams	Supervisor, field editor, and four interviewers who are the same sex as the respondents	See DHS
	Health technician(s) for biomarker testing	Separate measurer for anthropometry
Software package used for primary data processing	CSPro	CSPro
Imputation and data analysis	CSPro	CSPro→SPSS
Preparation of report	In-country report writing workshop	Regional workshops, in-country support
Technical assistance	Technical assistance visits by ICF International	Regional workshops, in-country support, regional coordinators
**Characteristics of nationally representative surveys conducted in 2011**		
Typical duration of fieldwork	3–6 months	2–4 months
Mean number of households	Around 15,000 households	Around 10,000 households
Average time between completion of data collection and release of the report	3 months for Preliminary Report, 10–12 months for Final Report	12–13 months for Final Report
**Other characteristics**		
Free public access to datasets	www.measuredhs.com	www.childinfo.org
Easy access to survey results	STATcompiler	MICS Compiler

AIS, AIDS Indicator Surveys; KIS, Key Indicator Surveys; MIS, Malaria Indicator Surveys; SPA, Service Provision Assessment Surveys.

**Table 2:** Differences between standard DHS and MICS protocols and their potential implications for coverage measurement.

Characteristics	DHS	MICS	Potential Implications for Coverage Measurement
**Sampling and survey design**			
Sample size per cluster	Rural: 30–40 women; Urban: 20–25 women	15–30 households	—
Construction of household rosters	All usual members of the household *plus* visitors who spent the previous night in the household. DHS tables on coverage measurement are based on de facto persons in the household (that is, persons who stayed in the household the previous night).	All usual members of the household (de jure household members) included.	De facto approach gives better representation of mobile populations. De jure approach is more consistent with selection probabilities based on censuses. Unlikely to lead to any bias, since response rates remain very high in both approaches.
Respondents for information about children less than 5 years of age	Biological mothers only except for anthropometric indicators and anaemia, which are collected for all children.	Mothers or primary caregivers of children under 5 living in the household.	Inclusion of caregivers means orphans and foster children are included in the samples for MNCH coverage estimates for MICS, and not for DHS. See Box 1 for implications for coverage measurement.
**Reference periods for selected MNCH coverage indicators**			
Skilled attendance at delivery	All births during the past 5 years	Last birth during the past 2 years	The advantage of a shorter reference period is that the coverage estimates refer to a more recent date; on the other hand, the sample size is reduced when the reference period is shorter, which increases the confidence intervals.
Antenatal care	Last birth during the past 5 years	Last birth during the past 2 years	See above
Tetanus toxoid	Last birth during the past 5 years	Last birth during the past 2 years	See above
Initial Breastfeeding	Last birth in the past 5 years	Last birth during the past 2 years	See above
Exclusive breastfeeding	Youngest child age 0–4 years living with the mother	All living children age 0–4 years	See above
Postnatal care	Last birth during the past 5 years	Last birth during the past 2 years	See above
Birth weight	All births in the past 5 years	Last birth during the past 2 years	See above

MNCH, maternal, newborn, and child health.

**Box 1. Orphans and Foster Children**

An important difference between MICS and DHS surveys is in the collection of information on children under 5. In MICS surveys, information on children under 5 is collected from mothers or primary caregivers of children under 5 in the household, making it possible to collect information on all children under 5 (including orphans and foster children), regardless of whether their biological mothers are in the same household. In DHS surveys, the bulk of information on children under 5 is collected from their biological mothers in the Woman's Questionnaire. Therefore, information on some coverage indicators is not collected for children under 5 who are orphaned or not living with their biological mothers. An exception is the collection of anthropometric measurements and biomarkers for all children under 5 in the household, regardless of the survival status or whereabouts of their biological mothers.

In a recent analysis of 12 DHS and MICS surveys in 12 countries (unpublished), we found that the inclusion or exclusion of orphaned and fostered children does not have a substantial influence on national estimates of undernutrition. In three of the surveys, there was more than a 5 percentage point difference in the prevalence of underweight between children living with their biological mothers and other children. However, in seven of the surveys, the difference was less than 2 percentage points. In all 12 surveys, the difference between the *national* estimate of the prevalence of underweight for all children under 5 and for children whose mothers were interviewed was negligible. The differentials may vary for other indicators, so additional research would be desirable.

Since their inception, DHS and MICS surveys have played an important role in shaping the global agenda on tracking coverage and in populating global databases. They have also influenced policies and intervention strategies. For example, DHS/MICS data are often used to establish targets in national economic and social development plans, to provide advocacy for programmes to improve women's and children's health, and to assist programmes in identifying target groups in most need of interventions. The role that these data play at the national and international level make it imperative that data quality is the foremost consideration when designing surveys and providing estimates of key indicators. In the following sections, therefore, we draw on DHS and MICS experience to highlight the challenges associated with measuring coverage through household surveys.

## Basic Principles and Survey Design

Valid measurement of coverage requires, first and foremost, representative population samples based on scientific probability sampling. We will discuss this essential aspect of coverage measurement in the next section of our review but first we will present three other key considerations that need to be taken into account when planning and conducting household surveys and when using their results.

First, some information is simply not amenable to collection through a household survey, because respondents do not know or cannot recall the required information. For example, a parent cannot know a child's birthweight if the child was not weighed at birth and is unlikely to remember the exact timing of tetanus toxoid vaccinations received over a lifetime. Decisions about what questions respondents can answer with acceptable accuracy are generally made by survey designers in consultation with technical experts; the research papers in this collection provide some of the first rigorous research assessing the validity of self-reported exposure to RMNCH interventions [17],[18],[25]–[28]. For coverage questions that cannot be answered through household surveys, alternative methods should be explored.

Second, survey interview length needs to be taken into account. In principle, a survey interview should be long enough to engage the respondent and obtain complete answers to the survey questions without rushing, but not so long that respondents become bored or frustrated. Completion of all questionnaires in a household averages around 2 hours for DHS and MICS surveys. Technical staff at DHS and MICS are concerned that the current protocols—especially when optional modules, biomarkers, and country-specific questions are included—are approaching lengths that adversely affect data quality. The need to keep survey interviews to a reasonable length is an important source of tension when discussing the addition of new questions with stakeholders.

Third, response rates are a concern in household surveys as low response rates can adversely affect the representativeness of the interviewed sample. Efforts need to be made to ensure that non-response is as low as possible. Non-response is well below 10% in DHS and MICS surveys for most countries. Even though DHS and MICS protocols specifically prohibit the substitution of households, response rates remain high thanks to training protocols emphasizing multiple revisits to households and close monitoring of response rates by field staff.

## Survey Sampling

The sample design determines the representativeness of household survey results, which is required to produce coverage estimates for the general population. Textbooks have been written about survey sampling (e.g., [29],[30]) and we will not attempt to reduce them to a few paragraphs here. We recommend strongly that all survey planning teams should include an experienced technical sampling expert. Moreover, it is important not to underestimate the time involved in doing sampling properly. DHS [31] and MICS [32] surveys adhere to the fundamentals of scientific sampling, including complete coverage of the target population, use of suitable sample sizes, the need to conduct a new household listing and pre-selection of sample households, and preparation of appropriate sample documentation. However, deviations from standard procedures are sometimes required owing to cost limitations and practical considerations, including security concerns. Importantly, coverage levels in household surveys that use uncontrolled non-probability sampling (such as quota sampling or purposive sampling) do not provide valid estimates of population coverage.

DHS and MICS sampling frames are limited to the population residing in fixed households, and exclude populations living in group quarters (such as military barracks, hospitals, and hotels) and persons living on the street. This is a potential source of bias, because those populations are likely to have different characteristics and health conditions from those living in households.

One very practical lesson learned through the DHS and MICS experience is that sample households should be selected in the central office, not in the field, if possible. Only preselected households should be eligible for interviewing, with no substitutions allowed during the fieldwork. This prevents interviewing teams from reducing their workload by avoiding listing or interviewing large or more remote households.

A key sampling question is how many households/individuals should be interviewed per sample cluster. DHS recommends a sample “take" of about 30–40 women per rural cluster and 20–25 women per urban cluster. MICS recommends 15–30 households per cluster, although detailed analyses of available budgets, logistical limitations, survey content, and information on sampling errors from previous MICS and DHS surveys are also undertaken on a case-by-case basis.

Currently, DHS and MICS surveys include around 15,000 and 10,000 households respectively, which is a sufficient sample size to produce statistically reliable estimates of most indicators at the national, urban–rural, and regional levels, but not at lower administrative levels, such as districts, slums, and small population groups. However, both survey programmes can oversample population groups or geographical areas to produce statistically valid estimates, when needed. Some DHS and MICS surveys have large enough sample sizes to produce estimates at lower administrative levels, but the added value of producing coverage estimates at these administrative levels with larger samples needs to be weighed against the added logistical and management challenges.

Unfortunately, the use of sampling weights is often misunderstood, and consequently misused or ignored. Some strata (such as urban areas or selected regions or provinces) are often oversampled to ensure that the final dataset includes enough cases to produce reliable results. In these cases, sampling weights need to be applied to account for varying design weights and non-response levels. Generally, analyses of survey data should use sampling weights calculated for each interviewed household or individual respondent. There is a lack of consensus, however, about whether weights should be used in multivariate analyses, and the decision is often based on the purpose of the analysis [33],[34].

## Comparability of Measurement over Time and between DHS and MICS

Tracking changes in RMNCH coverage over time is a valuable way to assess progress and is one of the most widespread applications of DHS/MICS data. But there are many pitfalls, and often coverage estimates are taken from survey reports and plotted to show changes without sufficient attention being paid to comparability. Notably, RMNCH coverage indicators change over time in response to modifications in policies and programmes and as lessons are learned about measurement and interpretation. Other papers in this collection report on multiple changes in indicators of diarrhoea case management since 1990 [19], more recent changes in treatment of childhood malaria [18], and postnatal care for mothers and infants [35]. Rather than abstracting coverage data from published survey reports, users should access the UNICEF childinfo.org database, in which all standard global indicators have been checked for comparability and recalculated where necessary. If users obtain DHS and MICS data files themselves to recalculate coverage indicators over time, care must be taken to use standard indicator definitions and appropriate sampling weights.

MICS and DHS collaborate closely and work through interagency processes to ensure that their survey tools are harmonized and comparable and their data can, therefore, be combined in global databases covering a large majority of developing countries. The article in this collection by Requejo, Newby, and Bryce highlights the importance of using standard methods to produce comparable coverage data across countries [36]. Differences between DHS and MICS surveys that may affect RMNCH coverage estimates are presented in Table 2, which also summarizes the evidence on the possible magnitude of the effects of these differences. Finally, as mentioned earlier, Box 1 highlights a particularly important difference between DHS and MICS surveys—their inclusion or exclusion of information on orphans and foster children.

## Challenges in Survey Implementation

Accumulated experience from the two survey programmes underscores the importance of incorporating quality control mechanisms at every step in the survey process. Some data quality steps are described elsewhere [36]; here we focus on several common field problems that could affect the quality of RMNCH coverage estimates.

One of the major field problem concerns is related to interviewer training and supervision. Table 1 presents information about how certain aspects of survey implementation are dealt with in MICS and DHS surveys. Both programmes have minimum requirements for selecting interviewers (at least a high school diploma). Moreover, interviewers are not directly involved in the management/provision of health services to avoid potential conflicts of interest.

Importantly, the MICS programme was originally intended to be a relatively quick and light exercise at the country level, with limited in-country technical support. The MICS programme was designed to produce the limited data needed to monitor progress towards the World Summit for Children Goals. Over time, however, concerns about quality issues associated with the expanded questionnaire length and increased sample sizes have led MICS to develop a technical support system that emphasizes the duration and content of interviewer training and field supervision, but also includes rigorous controls on data entry and checking. These changes bring the quality of MICS data more in line with that of DHS (which has always included substantial centralized technical support).

Another important quality assurance measure for both survey programmes concerns the entry of data for paper-based questionnaires simultaneously with the fieldwork. For both paper-based questionnaires and surveys conducted with personal data assistants (PDAs) or tablets, field check tables are produced while the fieldwork teams are still in the field, thereby making it possible to spot systematic errors in data collection and take measures to improve data quality.

However, despite meticulous attention to quality control, all household surveys are prone to quality problems, the most important of which we will now highlight. One of the most challenging aspects of implementing DHS and MICS protocols is how to ensure that these protocols are appropriately adapted to the country context. Despite the availability of technical guidelines and assistance, our experience suggests that problems frequently occur in this area. One clear example is the adaptation of response categories for diarrhoea treatment at home. The standard response category for “government-recommended home fluids" is often not customized to reflect country-specific recommendations, in many cases because of the lack of clear national policies or the long list of recommended fluids [19]. Another example is described by Hazel and colleagues in this collection [21] who show that coverage gains attributable to recently adopted strategies for treating childhood illness at the community level cannot be assessed unless the response options for care seeking are adapted to reflect the country-specific providers of care at the community level. Ensuring that standard survey protocols are adapted to specific country contexts is a continuing challenge, especially given the numerous topics included in each survey and the time and resources needed to adapt questionnaires in each country.

Another implementation challenge relates to what demographers working on child mortality refer to as the tendency for reports of child deaths to “heap" at certain ages, notably 12 months [37]. If this tendency is also true for reports of service utilisation and receipt of interventions, it could affect the accuracy of coverage estimates with time-bounded effectiveness, such as the timing of antenatal or postnatal care. We are not aware of any research that investigates the effects of age heaping in coverage measurement, and believe that this warrants further attention.

Finally, a related problem is the tendency for interviewers to “transfer" children to age groups (especially over 5 years of age) that exclude them from lengthy portions of the interview and therefore reduce interviewer workload [37]. Even if only some interviewers modify children's ages in this way occasionally, it can affect coverage estimates, especially if the children whose ages are changed systematically have different characteristics from other children. This is a continuing problem in some surveys despite serious attempts to minimize abuses. Age displacement may have more of an effect on fertility and mortality estimates than RMNCH estimates, but displacement remains a matter of concern overall.

## Conclusions

Large-scale, nationally representative household surveys are the primary source of data on RMNCH coverage. Despite efforts to improve routine information systems, surveys are likely to remain the primary source of data for many years to come. As we discuss in this review, it is essential that survey planners and consumers of survey results understand the strengths, limitations, and constraints of coverage measurements generated through household surveys and also appreciate the technical issues involved in sampling and quality control. Moreover, as our review and the other articles in this collection highlight, conducting household surveys that generate valid and reliable information on coverage is a complex exercise. We believe that the findings of current and future experimental studies will help to inform continuing efforts to improve coverage measurement in household surveys, particularly in the areas of improved question wording and interviewer training. Finally, we stress that calls for more and better data on coverage must be accompanied by sufficient resources and by an ongoing research programme to continue to improve and refine methods and analytical techniques.

**Key Points**

- DHS and MICS surveys are the principal source of national-level data on maternal, newborn, and child health indicators in low- and middle-income countries.
- Despite efforts to improve routine information systems, household surveys are likely to remain the primary source of population data for the foreseeable future.
- To analyse coverage estimates over time and across countries from household survey data, it is essential that similar survey methods and questions be employed to ensure comparability.
- The strengths and weaknesses of all data collection efforts need to be transparent and well understood by data users.
- DHS and MICS will continue to benefit from research findings to make further improvements in the collection of reproductive, maternal and child health coverage data.

## Abbreviations

AIS: AIDS Indicator Surveys
DHS: Demographic and Health Surveys
MICS: Multiple Indicator Cluster Surveys
MIS: Malaria Indicator Surveys
RMNCH: reproductive, maternal, newborn, and child health
